# Wear Performance Evaluation of Polymer Overlays on Engine Bearings

**DOI:** 10.3390/ma17153802

**Published:** 2024-08-01

**Authors:** Ismail Ozdemir, Bahattin Bulbul, Ugur Kiracbedel, Thomas Grund, Thomas Lampke

**Affiliations:** 1 Materials and Surface Engineering Group, Chemnitz University of Technology, Erfenschlager Str. 73, 09125 Chemnitz, Germany; 2 Eksel Bimetal Sintering and Casting Factory, Ankara Cad. No: 82A, 35100 Izmir, Turkey; bahattin@ekselbimetal.com.tr (B.B.);

**Keywords:** plain bearing, polymer overlay, copper-based bimetal, pin-on-disc, wear

## Abstract

Modern engine bearing materials encounter the challenge of functioning under conditions of mixed lubrication, low viscosity oils, downsizing, start–stop engines, potentially leading to metal-to-metal contact and, subsequently, premature bearing failure. In this work, two types of polymer overlays were applied to the bearing surface to compensate for extreme conditions, such as excessive loads and mixed lubrication. Two different polymer overlays, created through a curing process on a conventional engine bearing surface with an approximate thickness of 13 µm, were investigated for their friction and wear resistances under a 30 N load using a pin-on-disc setup. The results indicate that the newly developed polymer overlay (NDP, PAI-based coating) surface has a coefficient of friction (COF) of 0.155 and a wear volume loss of 0.010 cm^3^. In contrast, the currently used polymer overlay (CPO) in this field shows higher values with a COF of 0.378 and a wear volume loss of 0.024 cm^3^, which is significantly greater than that of the NDP. It was found that, in addition to accurately selecting the ratios of solid lubricants, polymer resins, and wear-resistant hard particle additives (metal powders, metal oxides, carbides, etc.) within the polymer coating, the effective presence of a transfer film providing low friction on the counter surface also played a crucial role.

## 1. Introduction

The escalating requirements for combustion engines, involving downsizing and reducing greenhouse gas emissions, have prompted innovations in engine bearing systems. These innovations are essential for preventing expensive engine downtime and catastrophic failures in engine components [1,2,3]. Under new legal regulations, the working conditions resulting in reduced fuel consumption and environmentally harmful waste gases indirectly lead to compulsory conditions on engine bearing materials [4,5]. These conditions, in turn, result in early damage or seizure of the bearing material under inadequate lubrication conditions that may lead to metal-to-metal contact [6]. One of the most commonly used bearing materials in practice is bronze alloys, which can easily be damaged by dry sliding wear resulting from inadequate lubrication conditions, especially during the start–stop function [7]. Therefore, new bearing materials need to possess mechanical and tribological properties capable of withstanding inadequate lubrication conditions [8]. For this purpose, electroplating, cold spray process [9], magnetron sputtering, and polymer thin-film coatings [10] have emerged as the most preferred methods, particularly in the production of self-lubricating bearings aimed at eliminating inadequate lubrication problems [1,11].

Among these applied techniques, thin polymer film coatings stand out because of their relatively low cost and the ease with which they can be sprayed onto the bearing surface as a permanent layer. Additionally, they exhibit superior sliding performance under certain conditions owing to their flexible geometric compatibility [12]. Polymer overlays where solid lubricants are dispersed in a resin binder are being developed in this sector [7]. When there is direct contact between a steel shaft and the bearing surface, the bearing surface overlay is damaged because of a lack of hydrodynamic lubrication. In the presence of a polymer-based overlay, wear is less likely to progress compared with conventional or even sputter-hardened bearing surfaces [10].

Fine dispersed hard particles, along with solid lubricant particles that are well bonded to the polymeric matrix, can effectively resist chemical attacks and consequently reduce wear under conditions of poor lubrication [13]. Molybdenum disulfide (MoS_2_), hexagonal boron nitride (h-BN), and graphite (C) have been added as the solid lubricant for resin overlays [14]. Under ideal conditions, these self-lubricating particles adhere to the metal surfaces during start–stop operation, forming a protective film that reduces friction.

However, with the increase in both thermal and mechanical load under poorly lubricated conditions, there may be a softening of the polymer matrix, resulting in the loss of the protective film at the mated surface, subsequently facilitating the transition from mild wear to severe wear. The tribological behavior of polymer thin film coatings, including their friction and wear mechanisms, is more complex compared to metals [15].

In addition to the behavior of the polymer matrix, it is also crucial for solid lubricants to have thermal stability at high temperatures, which is highly important in reducing friction. Furthermore, it has been experimentally demonstrated that various solid lubricants, when distributed at optimum ratios within the polymer matrix, improve seizure resistance compared with traditional polymer overlays [14].

For the binder, polyamide-imide (PAI) resin is used for its excellent thermal resistance, in order to bear the high temperature (up to 260 °C) environment in an engine. Because of its more elastic properties compared with other candidates [13], PAI resin enables excellent mechanical interlocking with adequately roughened lining surfaces, thereby preserving wear resistance even under abrasive wear conditions [16].

Various additives are utilized to optimize the polymer overlay strength to be competitive with higher tribological demands [7,13,17,18,19]. Wear-resistant hard particles (Al_2_O_3_, SiC, ZrO_2,_ etc.) and metal powders (Cu, Sn, Al, etc.) can be used for this purpose. Because polymer-based overlays are intended for permanent application on engine bearings, they have to endure the forces emitted into the bearing from powertrain components. They should, therefore, provide enough fatigue and wear resistance, embeddability [16], as well as seizure properties. In addition, polymer-based overlays act as a “permanent oil film” since single metallic overlays, presumed to possess sufficient friction, embeddability, adaptability, and conformability [5], fail to provide sufficient wear resistance, particularly under mixed lubrication conditions. The supplementary polymer layers on the top surface of engine bearings, named overlays, can serve as this “oil film” by separation of direct tribological contact between the two counter bodies of the tribological system, in particular, under stop–start conditions where minimal oil film thickness from active lubrication is present [20]. Polymer overlays help to reduce fuel consumption because they decrease friction and wear. They also help the amount of boundary and mixed friction lubrication regimes for highly loaded areas of the engine. Based on damage and surface analysis, it was reported that the wear performance of polymer overlay structures, particularly observed during start–stop operations, was attributed to the solid lubricants and fillers dispersed within the polymer matrix, forming a dense and well-bonded structure with the matrix [21].

Therefore, this study concentrates on the tribological characteristics of two types of polymer overlays containing different proportions of solid lubricants, metal powders, and wear-resistant hard particle additives. Polyamide-imide (PAI) is used as the matrix for the polymer coating study. The aim is to understand their impact on the wear performance of engine bearing materials utilized in real applications.

## 2. Materials and Methods 

### 2.1. Bearing Material, Polymeric Coating Deposition, and Characterization

**Figure 1:**
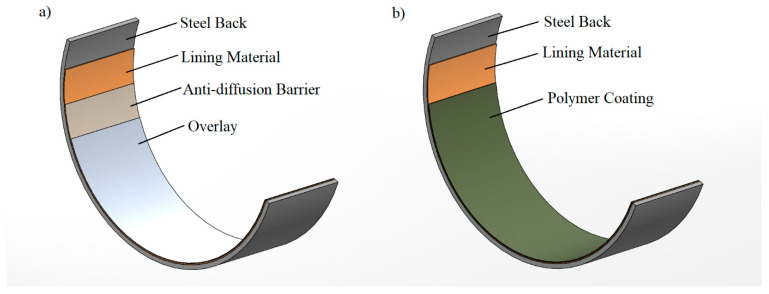
(**a**) Schematic illustration of the bearing structure of a conventional electroplated coated bearing and (**b**) the investigated type of polymer-coated bearing.

**Figure 2:**
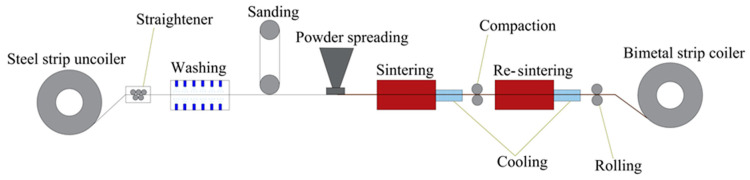
Copper-Based Sintering Bimetal production system.

The typical engine bearing material structure and polymer-coated engine bearing structure are given in Figure 1. Figure 1a shows a conventionally electroplated system, while Figure 1b shows the polymer-coated system addressed in this work. The bearing comprises a black steel strip that is sinter-coated using a bronze powder. To produce the sample material used in the present study, the bronze powder (for its composition, see Table 1) with a particle size under 150 µm was uniformly spread onto a moving steel strip. As the strip passed through a furnace in a reducing atmosphere, the bronze particles were sintered at between 800 and 900 °C (Sistem Teknik Industrial Furnaces, MBSF-525-047-120, Electrical Heater Controlled Atmosphere Furnace, Kocaeli, Turkey) and bonded to the steel layer with a thickness of approximately 500–600 µm. Subsequently, this bimetallic material was cold rolled to densify the liner alloy, achieving adequate bonding between the lining and steel back layers. This form of the Copper-Based Sintering Bimetal production system is shown in Figure 2. After bimetal production, the bronze lining surface underwent a cleaning and rinsing process in an ultrasonic alkaline bath at 60 °C before it was coated with the investigated polymer overlayers to eliminate oil, grease, and other residues. Afterward, it was dried in a dry furnace at 90 °C. Finally, the bearing surfaces were subjected to grit blasting using 220 mesh alumina particles, which resulted in an average Ra surface roughness of 0.90 µm. Finally, the polymer-based suspension was applied as overlayers onto the surface of the lining layer with a thickness of 12–16 µm, as illustrated in Figure 1b.

**Figure 3:**
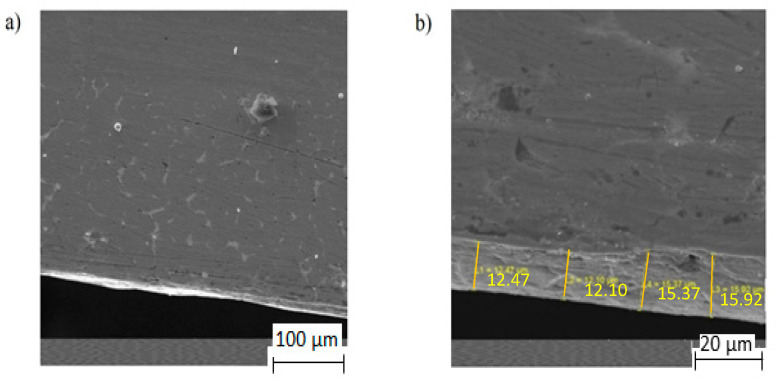
SEM images of the polymer overlay microstructure on the bronze bearing (**a**) at low (500×) and (**b**) high (2500×) magnifications.

Polymeric overlay materials were composed of a polyamide-imide (PAI) thermoplastic resin matrix blended with solid lubricants in a plate-like shape, such as MoS_2_ and graphite (D90:15 µm), up to 60 wt.%, and hard abrasives (D90:5 µm) containing metallic fillers (Al and Sn) distributed within a polymer matrix, up to 5 wt.%. Apart from these, some additives were added to the suspension in order to increase suspension spraying and leveling properties. Polyamide-imide was thinned with a solvent (NMP–N-methyl-pyrrolidone, Xylene) and then applied to the roughened bearing surface using a turntable automatic spraying gun. Following that, the materials underwent a curing process conducted between 190 and 230 °C for a period of approx. 1 h. To perform the microstructural observations, an optical microscope (OM, OLYMPUS/BX60M with LC Micro v2.2 image analyzer, Olympus Corporation, Tokyo, Japan) and a scanning electron microscope (SEM, Jeol JSM-6335F, Jeol, Tokyo, Japan) equipped with an EDS unit were used to determine the constituents of the powder and deposited layers. The corresponding cross-sections of the polymer overlay microstructure are shown in Figure 3. The higher magnification of the interface presented in Figure 3b demonstrates the robust adhesion of the polymer overlay to the metal surface.

### 2.2. Wear Tests

**Figure 4:**
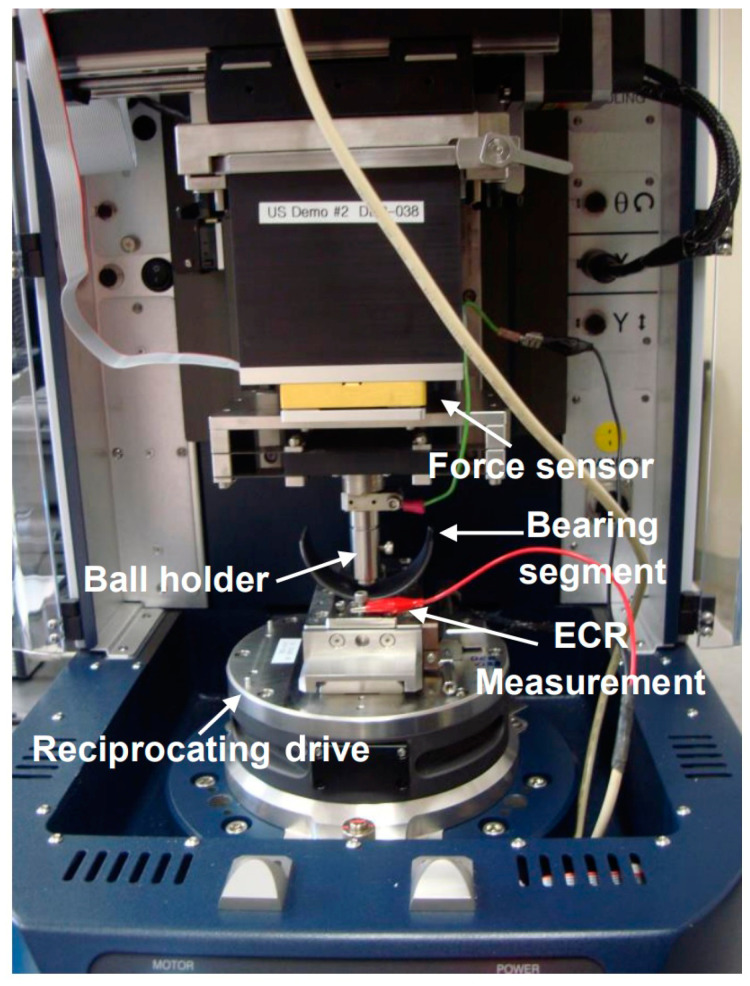
Wear test setup for a polymer-coated bearing segment.

To assess the performance of the application, polymer coatings with an average thickness of 13 µm were applied to 1585 LC-coded bearings. Subsequently, the bearings were subjected to wear under a 20–30 N load and moved in reciprocating motion over a stroke length of 10 mm at a frequency of 3 Hz in the wear test configuration (UMT Tribolab, Bruker, Ettlingen, Germany) illustrated in Figure 4. A bearing segment specimen was mounted on the reciprocating drive, and a 6.35 mm diameter 100Cr6 ball was installed under the force sensor (see Figure 3). The surface roughness of the counter ball was specified to be below 0.80 Ra. Wear tests were performed using reciprocating motion, with the speed varying from 0.002 to 10 mm/s, and no lubrication was employed during the tests.

Fx, Fz, Z-encoder (Z), and electrical contact resistance (R1) data were recorded during each test. The Z-encoder specifically measured the wear depth by precisely tracking the vertical displacement of the surface along the Z-axis. The coefficient of friction (COF) was obtained as the ratio of Fx and Fz. To assess the performance of the polymer top coating on bronze bearings in practical application, electrical contact resistance (ECR) was measured during the wear test, as shown in the arrangement in Figure 4. Throughout the wear test, ECR measurements were taken on a bearing surface without a polymer top coating, serving as a reference. The abrupt drop point recorded during the tests was evaluated as the point exceeding the durability limit of the polymer top coating. The wear rate was calculated from the Z data. The worn polymer overlay surfaces were examined using a 3D optical profilometer (ContourX-200, Bruker, Bremen, Germany) to analyze their wear behaviors. Once both the worn and intact regions of the sample were scanned using a 3D profilometer, a reference plane was established based on the intact surface, and volume loss was measured by comparing this reference plane to the worn surface.

## 3. Results and Discussion

### Wear Behavior of the Polymer Overlay

**Figure 5:**
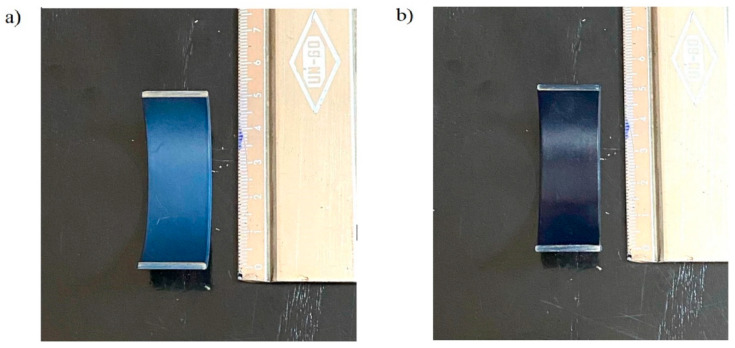
Macroscopic view of the tested polymer overlays: (**a**) the reference material (commercial), and (**b**) the newly developed material sprayed onto the bronze bearing surface.

A macroscopic overview of the polymeric overlay used in the bearing shell is given in Figure 5. As shown in the figure, a smooth polymer coating, similar to the reference material, was successfully achieved on the roughened bearing surface.

**Figure 6:**
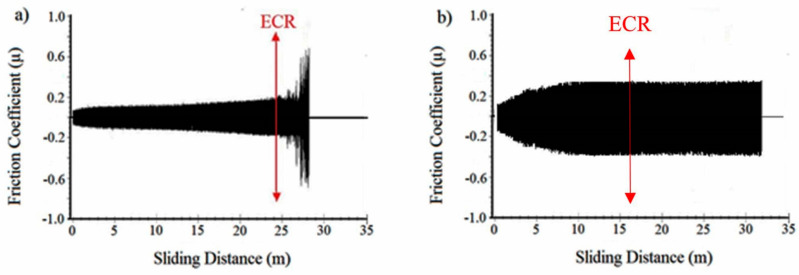
The variation in the coefficient of friction for the reference coating (**a**) and the newly developed polymer overlay coating (**b**) under a 20 N applied wear load for a longer sliding distance.

To determine the behavior of polymer coatings under high loads and extended sliding distances, the samples were subjected to wear tests under 20 N and 30 N loads until polymeric overlay damage or failure occurred. In Figure 6, the variation in the coefficient of friction is provided for samples subjected to a lower wear load, 20 N, and cycled until polymer overlay failure. It was found that in the reference material, polymer top coating damage initiation was detected at approximately 240 m of sliding distance (indicated by the red arrow in Figure 6a) and thereafter, the coefficient of friction rapidly increased, leading to the delamination of the coating from the surface. In the NDP coating, on the other hand, damage in the polymer top coating was observed at 165 m of sliding distance. However, while the coefficient of friction remained stable, the polymer overlay continued to protect the bearing material. This could be attributed to the NDP coating exhibiting higher adaptability during the running-in period, maintaining a low coefficient of friction despite longer sliding distances. The relatively good performance of polymer coatings in the damage analysis of various thin coatings on the bearing surface is attributed to the solid lubricants embedded in the polymer matrix. This solid lubricant, assisted by other particles, i.e., hard abrasive and metallic powders, within the polymer matrix, enhances the load-carrying capacity by adhering to the mating surface and facilitates the dissipation of heat generated during wear away from the system [1]. In another study, the effect of using solid lubricants with different sliding properties within the polymer matrix on the performance of polymer coatings was investigated. The study demonstrated that by utilizing solid lubricants in optimal ratios within the polymer matrix, new polymer thin film coatings were developed without damage even under poor lubrication conditions and extended test durations [14]. Additionally, the lifespan of the engine bearing was significantly increased through a well-designed and combined h-BN/C coating, resulting in a low coefficient of friction (COF) because of self-lubricating and highly adhesive films on the mating surfaces at both room and elevated temperatures [2]. Therefore, maintaining a low COF value of the NDP polymer throughout the test indicates that serious synergistic damage effects on the bearing surface did not develop compared with the ECR value.

**Figure 7:**
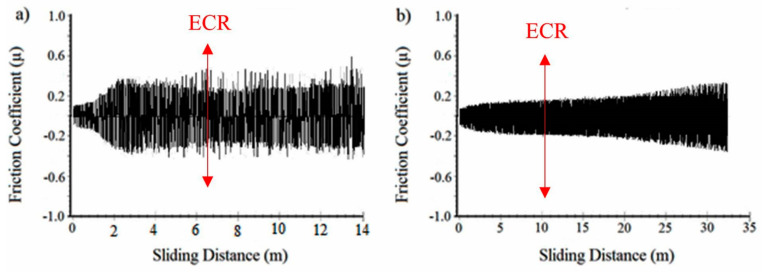
The variation in the coefficient of friction and ECR measurement (indicated by the red arrow) for the reference coating (**a**) and the NDP overlay (**b**) material tested under 30 N load.

At high load, 30 N, in the wear test, both the reference coating and the NDP coating began to delaminate from their horizontal surfaces at longer wear distances. Specifically, a sudden drop in the ECR value was observed after 6.5 m for the reference coating and 10 m for the NDP coating (see Figure 7).

Moreover, taking into account the additional wear data presented in Table 2, the average COF for the reference coating was measured to be 2.5 times greater than that of the NDP polymer top coating. As the load increases, the polymer surface is subjected to higher contact pressures, which can lead to high local temperatures during this contact, causing delamination stresses and resulting in premature damage. Therefore, the quality of the interfacial bonding of particles in the polymer matrix is very important to compensate for thermal mismatch and provide low friction in contact under heavy load.

According to previous studies, the superior wear resistance exhibited by polymer coatings compared with conventional lining materials is associated with the homogeneous distribution of hard particles, metallic components, and lubricating fillers within the polymer matrix, as well as their alignment along the loading direction, and the rapid removal of heat from the mating surface [10]. It was also recently shown that as the hard reinforcements within a polymer exceed the optimum value and agglomeration increases, abrasive wear and a high coefficient of friction cause the polymer coating to rapidly lose its function, particularly at high temperatures [17].

The relatively lower coefficient of friction in the NDP coating resulted in less wear loss compared with the reference coating. According to the data in Table 2, the reference polymer coating exhibited significantly higher volumetric wear loss compared with the NDP polymer top coating, more than double that of the NDP polymer top coating.

Accordingly, the NDP polymer coating exhibited both a low COF and reduced volumetric wear loss, indicating the presence of distinct wear mechanisms during the experiment. It was reported that especially under mixed-friction conditions, the better tribological performance and adaptability of the polymer layer depended on several factors. These factors included hardening temperature, filler materials such as solid lubricants, and the interaction between the polymer layer and the bearing material in the presence of dirt or trapped particles [15]. Moreover, this study demonstrated that the shape of solid lubricants is as critical as their ratio. Improvement in friction resistance was achieved when the MoS_2_-to-graphite ratio was maintained between 1.5:1 and 2.5:1, highlighting the significant influence of both parameters on tribological performance.

**Figure 8:**
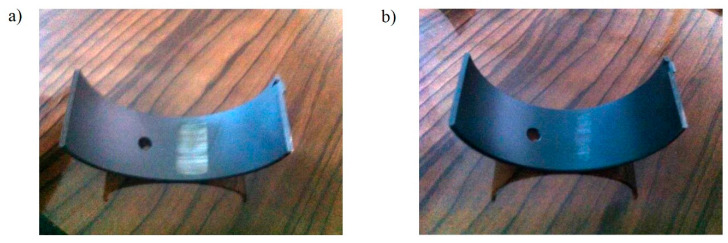
The macroscopic appearance of the worn surfaces of the reference polymer (**a**) and the NDP overlay (**b**) after the wear test under a 30 N load.

As observed in the macro images provided in Figure 8, it is evident that the bearing segments of both coatings exhibited a gradual removal of the overlay during the test. However, it was more pronounced for the reference coating, as the overlay detached from the lining surface.

The main purpose of polymeric layers on the bearing surface is to enhance not only the running-in behavior but also the geometrical adaptability. However, in comparison with the reference polymer, the NDP overlay adhered to the shaft surface or formed a transfer film during the running-in period, resulting in a reduction in the COF and wear rate. It was demonstrated that the development of a transfer film on the countersurface was a critical factor in polymer tribological performance. Furthermore, such a uniform film, which prevented scuffing or seizure, was generated by entrapping polymer wear debris within the asperities of the countersurface and progressively encasing them [22].

**Figure 9:**
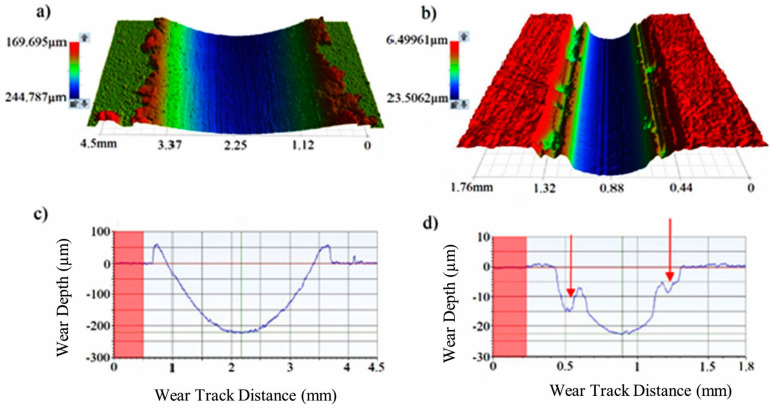
Three-dimensional wear track images of the reference polymer (**a**) and the NDP overlay (**b**) after wear tests under 30 N also displaying the depth of worn surfaces at (**c**) and notable breaks in the side walls of the wear path (red arrows) (**d**), respectively.

In a previous study, it was reported that the reduction in wear was associated with the use of an appropriate solid lubricant that forms a compatible and low-friction transfer film on the counter surface [2]. The three-dimensional wear track images obtained to gain more insight into the wear behavior of polymer top coatings after a wear test carried out under a 30 N load are presented in Figure 9.

As seen in the image, the wear track of the reference polymer deformed relatively homogeneously. Simultaneously, it is evident that under the applied load, the polymer layer formed an outward deformation lip beyond the wear track, leading to the recorded highest wear depth, approximately 220 µm. In contrast, the NDP polymer overlay exhibited a significantly different wear behavior, demonstrating a narrow and cohesive wear pattern compared with the wear track of the reference polymer. Additionally, it displayed resistance to the abrasive counter material, resulting in localized fractures, as indicated by the arrow on the wear wall. Furthermore, it should be noted that the patches adhering to the counter surface by detaching from the polymer surface reduced the wear depth by minimizing abrasion between the two surfaces. The character and compatibility of pre-deposited transfer layers under dry sliding conditions in various polymer-bearing materials have been demonstrated to significantly impact wear characteristics, particularly in terms of low friction and wear volume loss [23]. As highlighted in a recent study, however, despite the recognized importance of transfer films in reducing friction and wear in dry sliding conditions involving polymers and metals, their kinetics, stability, and real-time correlation with friction remain poorly understood. This is primarily due to the lack of time-resolved quantitative data on transfer film formation [24].

**Figure 10:**
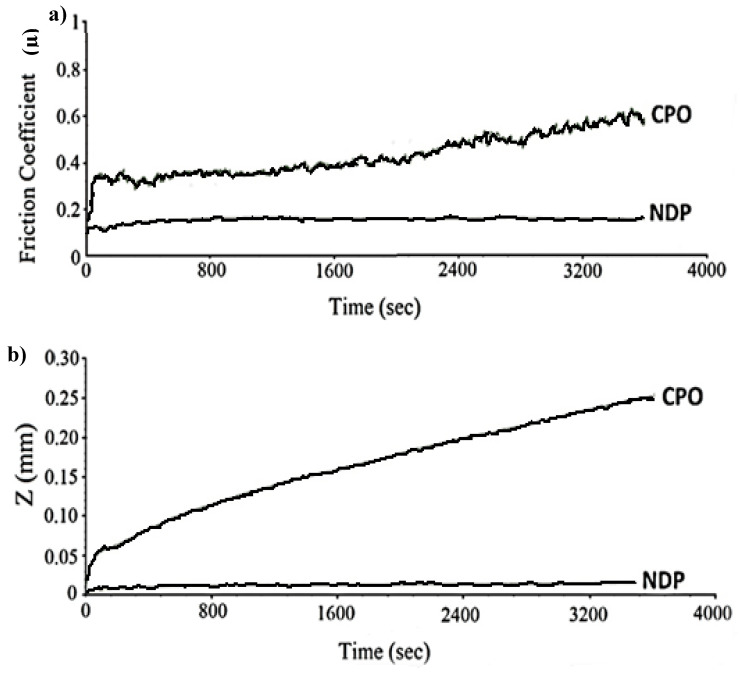
The variation in the COF values (**a**) and linear wear loss (**b**) tested under 30 N.

Upon encountering abrasive hard ball surfaces, these comparatively pliable polymer-coated exteriors manifested a COF not exceeding 0.1. Nevertheless, with prolonged wear, subsequent to a specific threshold, the COF attained stability, signifying substantial wear resistance (see Figure 10). This finding was attributed to the gradual settling of the ball onto the surface over time, wherein the coating interacts harmoniously with the underlying material, signifying a successfully attained conformability between the mating surfaces.

**Figure 11:**
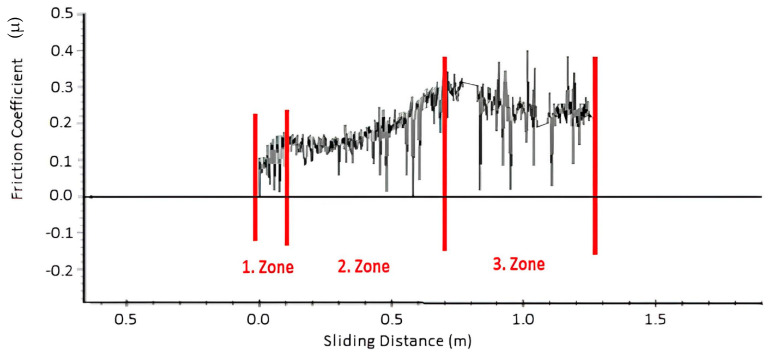
The change in the COF as a function of wear distance when the reference polymer coating initially comes into contact with a ball under a 30 N load (1st zone), depicting the region where it functions in harmony with the ball (2nd zone) and the point at which it completely separates from the wear surface (3rd zone).

This observation is evident from the linear wear loss, indicating that in the reference polymer top coating, the amount of wear increases with wear duration, while it remains constant in the NDP polymer. As illustrated in the friction graph given in Figure 11 for the reference polymer coating, during the initial stages of wear (first zone), the ball, influenced by the wear load, deforms the polymer coating to increase the contact surface. In the second zone, the polymer coating collaborates with the ball for a while to stabilize friction. However, with increasing wear distance, the wear resistance of the coating decreases, leading to an increase in the COF value. As a result, a rapid increase in wear loss leads to the disappearance of the polymer coating as the opposing material directly contacts the bearing material (third zone). This could be attributed to the interfacial breakdown between the polymer matrix and hard reinforcements, particularly the removal of hard particles, which facilitated abrasive wear because of the inadequate embeddability of the PAI-based overlay [16]. Moreover, it was demonstrated that the incorporation of solid lubricants enhanced the tribological properties of a coating by modifying the polymer matrix [25].

## 4. Conclusions

Two types of polymer overlays, formed through a curing process on a traditional engine bronze bearing surface with an estimated thickness of 13 µm, were studied regarding their friction and wear properties. The investigation involved applying normal loads ranging from 20 N to 30 N using a pin-on-disc setup. The main results of this study can be summarized as follows:

- After the pin-on-disc test, the NDP overlay demonstrated significantly lower friction with a coefficient of 0.155 and a reduced wear volume loss of 0.010 cm^3^. In contrast, the reference polymer surface exhibited higher friction with a coefficient of 0.378 and a wear volume loss of 0.024 cm^3^, which was 140% greater than that of the NDP.
- Because of the relatively lower friction exhibited by the NDP coating compared with the reference coating under varying loads, the NDP overlay can contribute to reducing fuel consumption in the engine.
- In the 30 N wear test, the reference polymer exhibited the highest wear depth (220 µm), whereas the NDP coating demonstrated a narrow and cohesive wear pattern, indicating low and limited abrasive wear occurred based on the wear track results.
- NDP coating protected the counterpart surface under metal-to-metal contact situations. This is beneficial for crankshaft surfaces during the engine run-in period.
- The results of the ECR test indicated that it could be a useful indicator for the loss of surface protection of polymer coatings as they wear out during the running-in period of engine bearings.

## Figures and Tables

**Table 1:** Bronze powders used for the lining alloy.

Alloy	Chemical Composition			
	Copper	Tin (wt.%)	Lead (wt.%)	Nickel (wt.%)
SAE49 (Copper–Tin–Lead Alloy)	Rest	0.7–2.0	19.0–27.0	max. 0.2
SAE794 (Copper–Tin–Lead Alloy)	Rest	3.0–4.5	19.0–27.0	max. 0.2
CuSn8Ni (Copper–Tin–Nickel Alloy)	Rest	7.0–9.0	<0.1	0.7–1.3

**Table 2:** Wear data obtained under a 30 N wear load for the reference and NDP overlay.

Specimen	Coating Thickness (µm)	Time to ECR Drop (s)	Mean COF (µ)	Linear Wear Rate (mm/h)	Wear Volume Loss (cm^3^)
Reference polymer overlay	13	7	0.378	0.253	0.024
NDP polymer overlay	13	13	0.155	0.017	0.010

## Data Availability

The original contributions presented in the study are included in the article, further inquiries can be directed to the corresponding author.
